# 2658. Mortality among children hospitalized with suspected pneumonia during the COVID-19 pandemic in India (PREVAIL study)

**DOI:** 10.1093/ofid/ofad500.2269

**Published:** 2023-11-27

**Authors:** Yangyupei Yang, Maria D Knoll, Anurag Agarwal, Vikas Manchanda, Melissa Higdon, Rajeshwar Dayal, Arti Agarwal, Deepti Chaurasia, Jyotsna Shrivastav, Dhulika Dhingra, Shariqa Qureshi, Rajni Sharma, Richa Choudhary, Subhashish Bhattacharyya, Sudip Saha, Kayur Mehta, Anita Shet

**Affiliations:** Johns Hopkins Bloomberg School of Public Health, Baltimore, Maryland; International Vaccine Access Center, Johns Hopkins University, Baltimore, Maryland; Maulana Azad Medical College, Delhi, Delhi, India; Maulana Azad Medical College, Delhi, Delhi, India; International Vaccine Access Center, Johns Hopkins University, Baltimore, Maryland; Sarojini Naidu Medical College, Agra, Uttar Pradesh, India; Sarojini Naidu Medical College, Agra, Uttar Pradesh, India; Gandhi Medical College, Bhopal, Bhopal, Madhya Pradesh, India; Gandhi Medical College, Bhopal, Madhya Pradesh, India; Chacha Nehru Bal Chikitsalaya, New Delhi, Delhi, India; Chacha Nehru Bal Chikitsalaya, New Delhi, Delhi, India; Sawai Mann Singh Medical College, Jaipur, Rajasthan, India; Sawai Mann Singh Medical College, Jaipur, Rajasthan, India; Chittaranjan Seva Sadan and Shishu Sadan Hospital, Kolkata, West Bengal, India; Chittaranjan Seva Sadan and Shishu Sadan Hospital, Kolkata, West Bengal, India; International Vaccine Access Center, Johns Hopkins University, Baltimore, Maryland; International Vaccine Access Center, Johns Hopkins University, Baltimore, Maryland

## Abstract

**Background:**

COVID-19 has indirectly led to excess deaths attributable to other illnesses. Pre-pandemic studies in India have shown mortality rates of 3-12% amongst young children hospitalized with suspected pneumonia. Here, we describe mortality among young children hospitalized with pneumonia during the COVID-19 pandemic.

**Methods:**

Children aged 1-35 months hospitalized with suspected pneumonia were enrolled prospectively across six sites in India. Demographic and clinical information were obtained, and children were followed until 90 days after hospital discharge.

**Results:**

Between October 2020 and November 2022, 4,517 children with suspected pneumonia were enrolled and 4,443 (98.4%) with known mortality status were included in this analysis. Monthly average enrollment was 104 before and during the SARS-CoV-2 peak Delta period (April–June 2021) and 213 post-peak-Delta period (July 2021–November 2022). 71% had WHO-defined severe pneumonia, 26% had SpO2< 90% at admission, 39 of 3000 tested were SARS-CoV-2 positive, and 17% left against medical advice (LAMA). Of 723 total deaths (case fatality ratio (CFR)=16%), 42% were detected in-hospital, 23% occurred within 48 hours after discharge/LAMA, and 35% occurred 2-90 days after discharge/LAMA. CFR was higher among children before and during than post Delta peak (28% vs. 11%, p< 0.001) but average number of deaths per month was similar (28.2 vs. 27.6). Mortality was higher in LAMA cases (32% vs. 5.3%, p< 0.001) and accounted for most (57%) post-hospitalization deaths.

**Conclusion:**

A high CFR (16%) was observed for young children hospitalized for pneumonia in India, with 58% of deaths occurring after discharge. Deaths were higher during the-Delta and Delta wave wave of the COVID-19 pandemic. Although very few children had COVID-19, pandemic-related restrictions and late presentation to healthcare may have contributed to high mortality from pneumonia.

**Disclosures:**

**All Authors**: No reported disclosures

